# Splicing arrays reveal novel RBM10 targets, including SMN2 pre-mRNA

**DOI:** 10.1186/s12867-017-0096-x

**Published:** 2017-07-20

**Authors:** Leslie C. Sutherland, Philippe Thibault, Mathieu Durand, Elvy Lapointe, Jose M. Knee, Ariane Beauvais, Irina Kalatskaya, Sarah C. Hunt, Julie J. Loiselle, Justin G. Roy, Sarah J. Tessier, Gustavo Ybazeta, Lincoln Stein, Rashmi Kothary, Roscoe Klinck, Benoit Chabot

**Affiliations:** 1 Health Sciences North Research Institute, Sudbury, ON P3E 5J1 Canada; 2 Biomolecular Sciences Program, Laurentian University, Sudbury, ON P3E 2C6 Canada; 3 Department of Chemistry and Biochemistry, Laurentian University, Sudbury, ON P3E 2C6 Canada; 4 RNomics Platform of Université de Sherbrooke, Sherbrooke, QC Canada; 5 Ottawa Hospital Research Institute, Ottawa, ON K1H 8L6 Canada; 6 Ontario Institute for Cancer Research, MaRS Centre, Toronto, ON M5G 0A3 Canada; 7 Departments of Medicine and of Cellular and Molecular Medicine, University of Ottawa, Ottawa, ON K1H 8M5 Canada; 8 Département de Microbiologie et d’infectiologie, Faculté de Médecine et des Sciences de la Santé, Université de Sherbrooke, Sherbrooke, QC Canada

**Keywords:** RBM10, SMN2, Alternative splicing, Splicing array, Spinal muscular atrophy, Cancer

## Abstract

**Background:**

RBM10 is an RNA binding protein involved in message stabilization and alternative splicing regulation. The objective of the research described herein was to identify novel targets of RBM10-regulated splicing. To accomplish this, we downregulated RBM10 in human cell lines, using small interfering RNAs, then monitored alternative splicing, using a reverse transcription-PCR screening platform.

**Results:**

RBM10 knockdown (KD) provoked alterations in splicing events in 10–20% of the pre-mRNAs, most of which had not been previously identified as RBM10 targets. Hierarchical clustering of the genes affected by RBM10 KD revealed good conservation of alternative exon inclusion or exclusion across cell lines. Pathway annotation showed *RAS* signaling to be most affected by RBM10 KD. Of particular interest was the finding that splicing of *SMN* pre-mRNA, encoding the survival of motor neuron (SMN) protein, was influenced by RBM10 KD. Inhibition of RBM10 resulted in preferential expression of the full-length, exon 7 retaining, *SMN* transcript in four cancer cell lines and one normal skin fibroblast cell line. SMN protein is expressed from two genes, *SMN1* and *SMN2*, but the *SMN1* gene is homozygously disrupted in people with spinal muscular atrophy; as a consequence, all of the SMN that is expressed in people with this disease is from the *SMN2* gene. Expression analyses using primary fibroblasts from control, carrier and spinal muscle atrophy donors demonstrated that RBM10 KD resulted in preferential expression of the full-length, exon 7 retaining, *SMN2* transcript. At the protein level, upregulation of the full-length SMN2 was also observed. Re-expression of RBM10, in a stable RBM10 KD cancer cell line, correlated with a reversion of the KD effect, demonstrating specificity.

**Conclusion:**

Our work has not only expanded the number of pre-mRNA targets for RBM10, but identified RBM10 as a novel regulator of *SMN2* alternative inclusion.

**Electronic supplementary material:**

The online version of this article (doi:10.1186/s12867-017-0096-x) contains supplementary material, which is available to authorized users.

## Background

RNA Binding Motif 10 (RBM10) is an RNA binding protein that has an arginine-rich amino terminal, two RNA Recognition Motifs (RRMs), a C_2_H_2_ zinc finger, a RanBP2 zinc finger, an OCtamer REpeat element and a G-patch [1]. It binds to a number of RNA targets, including sequences encoding NUMB [2], RBM5 and other RNA binding proteins [3]. Functionally, overexpressed RBM10 causes apoptosis, and low levels of RBM10 are associated with decreased sensitivity to an apoptogenic stimulus [4], and increased colony formation [2]. Mechanistically, RBM10 is involved in mRNA stabilization [5] and regulation of alternative splicing [2, 3, 6–8].

From a systemic point of view, expression of *RBM10* is vital for normal embryonic development. Null mutations in *RBM10* affect development of the brain, face, heart, lungs, kidneys, limbs and central nervous system during early embryogenesis: the most aggressive phenotypic manifestation of null *RBM10* mutation is TARP syndrome (comprising Talipes equinovarus, atrial septal defect, Robin sequence and cleft palate), an X-linked recessive disorder that results in early mortality—predominantly as a result of heart conduction defects [9, 10]. Changes in alternative splicing of the pre-mRNA from key target genes may be how mutated *RBM10* contributes to TARP syndrome [3]. Expression of RBM10 is also important for tumor suppression [11], and reduced expression has been observed in pancreatic cancer [12] and non-small cell lung carcinoma [13]. Paradoxically, expression of RBM10 (like its paralogue, the tumor suppressor RBM5) is elevated in some cancers, such as multiple endocrine neoplasia type 1 [14], breast cancers [15], liver and colon cancers [16] and some metastatic melanomas [17]. Elevated expression may be related to upregulation of pro-apoptotic signaling pathways triggered by cancer-related abnormalities, such as uncontrolled proliferation, but the definitive reason for this elevation is unknown.

Since RBM10 is a tumour suppressor, and the regulation of alternative splicing is an important process that is deregulated in cancer [18–20], we set out to identify novel RBM10-regulated alternative splicing events using cancer cell lines. We used siRNAs to inhibit RBM10 expression in two ovarian, two breast and one prostate cancer cell line(s), as well as one non-cancer cell line. We examined arrays of alternative splicing events (ASEs) using a reverse transcription-PCR screening platform [21]. An *SMN* ASE was the focus of more detailed expression analyses, at both the RNA and protein levels, in primary fibroblasts and a breast cancer cell line with stable RBM10 knockdown (KD). Our results indicated that RBM10 was capable of regulating the expression of *SMN2* RNA and protein, RBM10 inhibition correlating with increased expression of the full-length *SMN2* RNA and protein. These results suggest that RBM10 functions as an inhibitor of *SMN2* exon 7 splicing.

## Methods

### Cell lines

Epithelial adenocarcinoma cell lines were from breast (MCF-7 and MDA-MB-231), ovary (SKOV-3 and NIH:OVCAR-3) and prostate (PC-3), and the fibroblastic normal cell line was from skin (BJT). Cells were grown in Dulbecco modified eagle medium (DMEM) (MCF-7, MDA-MB-231, SKOV-3, NIH:OVCAR-3), Ham F-12 medium (PC-3) or alpha-minimal essential medium (BJT), all supplemented with 10% fetal bovine serum (FBS). The MCF-7 cell line with stable RBM10 KD was generated as previously described [4].

### Splicing arrays

In the cell lines used for the splicing arrays, RBM10 KD was achieved using two distinct and non-overlapping siRNAs: RBM10 siRNA1 (exon 7) 5′-AAG GUG UCG AUG CAC UAC A-3′; RBM10 siRNA2 (exon 23) 5′-GCA UUG UAA CGC CUA UCG A-3′, purchased from Dharmacon (Cedarlane, Burlington, Canada). siRNAs were transiently transfected as previously described [21]. RBM10 expression inhibition (KD) was assessed for the splicing arrays by immunoblotting, 96 h post-transfection, using a rabbit anti-RBM10 primary antibody (Cedarlane Laboratories Ltd; ARP30103), a rabbit anti-ACTIN primary antibody (Sigma; A2066) (for protein normalization) and a goat HRP-conjugated anti-rabbit secondary antibody (GE Life Sciences; NA934).

RT-PCR assays for the siRNA experiments were carried out as previously described [21]. The data can be accessed at http://rnomics.med.usherbrooke.ca/palace?purl=pcrreactiongroup/list/299. The list of genes and primers can be found on-line (http://rnomics.med.usherbrooke.ca/palace?purl=data/related/2495) (Additional file 1). Visualization and analysis of amplified products was carried out using the LabChip HT DNA Assay on an automated microfluidic station (Caliper, Hopkinton, MA, USA).

### Alternative splicing analysis

Primer sequences used in RT-PCR assays to (a) confirm the ASEs observed in the splicing arrays and (b) investigate SMN alternative splicing in the fibroblasts, were the same as those used in the splicing arrays (AlphaDNA, Montreal, PQ). *RBM10* primers used to confirm KD in fibroblasts and MCF-7 cells (RBM10F and RBM10v1/v2R), and control *ACTIN* (actinF and actinR) primers, were as previously described [4, 15]. PCR amplification conditions for *RBM10* were as previously described [15]. PCR amplification conditions for *SMN2* and actin were 95 °C 4 min, followed by 40 cycles (*SMN*) or 25 cycles (*ACTIN*) of 95 °C 30 s, 59 °C (*SMN*) or 67 °C (*ACTIN*) 30 s, 72 °C 30 s, followed by 72 °C for 10 min.

### Hierarchical clustering analysis

Genes with a change in at least one cell line were subjected to two-way unsupervised hierarchical clustering using the R package ‘pheatmap’. Hierarchical clustering analysis was done using Euclidean distance and either average or ward.D linkage. Pathway enrichment analysis was carried out using an in-house tool based on pathways downloaded from Reactome (release 55), the latest version of KEGG (November 2015), BioCarte (January 2012) and Panther (version 3.0.1).

### Human fibroblast analyses

Primary human fibroblasts: control (GM08333), carrier (NA03814) and SMA Type 1 (NA03813) (Coriell Institute for Medical Research, Camden, New Jersey, USA) were grown in DMEM supplemented with 15% FBS. All media and supplements were purchased from Life Technologies (Burlington, Canada). The passage numbers of fibroblasts used in the transient RBM10 KD studies were: control 6–9, carrier 11–14, and SMA Type 1 8–12. RBM10 KD was achieved using siRNA2 (to exon 23) (Thermo Fisher Scientific, Waltham, MA, USA).

### Immunoblotting

Protein expression in fibroblasts was examined by Western blotting. A mouse anti-SMN antibody was used (1:20,000 dilution; Cat. No. 610647, BD Biosciences, Mississauga, Canada) with either (1) a near infra-red fluorescent dye (IRDye 680RD) conjugated goat anti-mouse IgG (Li-COR, Lincoln, NE, USA) for in-gel probing using the Odyssey CLx Imaging System, or (2) an HRP-conjugated goat anti-mouse IgG (1:10,000 dilution, Santa-Cruz Biotechnology, Inc., Dallas, Texas, USA) for film-captured chemiluminescence and quantification using the densitometry tool on the AlphaEaseFC Gel Imaging System (Alpha Innotech, San Leandro, CA, USA). A rabbit anti-RBM10 antibody (1:5000 dilution, ProteinTech, Rosemont, IL, USA), and a rabbit anti-β-ACTIN antibody (1:10,000 dilution; Novus Biologicals, Littleton, CO, USA), were used with a goat anti-rabbit HRP-conjugated secondary antibody (1:10,000 dilution; Santa-Cruz Biotechnology).

## Results

To identify novel alternative splicing events associated with RBM10 inhibition we used splicing arrays that incorporated RNA from cell lines either mock transfected or individually transfected with two non-overlapping RBM10-specific siRNAs, and primers with the potential to yield two amplicons of between 150 and ~700 bp (for adequate resolution by microfluidic analysis). For each ASE, a percent splicing index change (Δψ) was calculated as the shift in splice site selection based on the difference between the ψ obtained with a given siRNA and the ψ obtained from the control transfection performed on the same day. A change was defined as the Δψ of eight percentage points or more, a threshold value that was based on [Z]-scores for control reactions performed with the ASEs in previous studies [21]. We added the further requirement that both siRNAs must produce a shift in the same direction (i.e., exon exclusion or exon inclusion).

### 29% of 96 cancer-related ASEs were changed in one or more of five cancer cell lines upon RBM10 KD

**Fig. 1:**
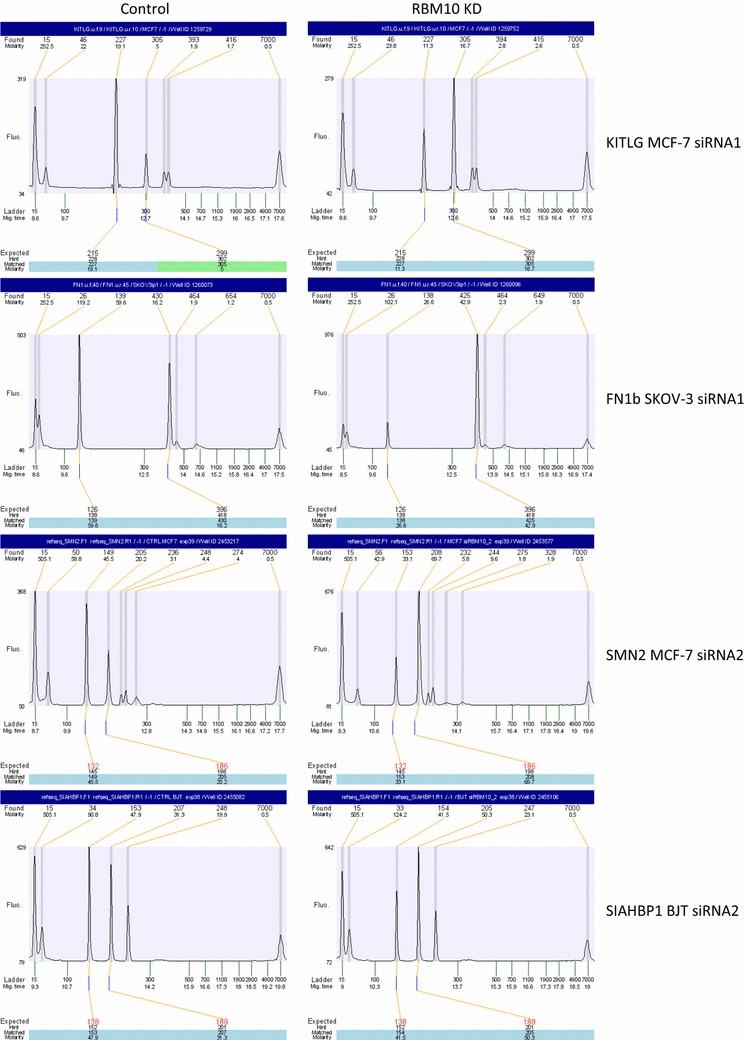
Electropherogram results for KITLG and FN1b ASEs from Array-96 and for SMN2 and SIAHBP1 ASEs from Array-191. The electropherograms on the *left* are from the control transfections using scrambled siRNA, while the electropherograms on the *right* are from the RBM10 KD transfections using the siRNA indicated. *Each row* represents the results from the cell line indicated. The “expected” amplicon size is shown *below* the electropherogram, while the “found” amplicon size is shown *above*

We initially examined predominantly cancer-associated genes known to have ASEs that change between normal and cancer tissues. This initial screen consisted of 96 ASEs from 92 genes (see below for link to data with list of genes). RNA from five cancer cell lines (MCF-7, MDA-MB-231, SKOV-3, OVCAR-3 and PC-3) was used. Representative electropherogram data, from the capillary electrophoresis of specific ASEs in individual cell lines with one siRNA, can be seen in Fig. 1.

**Fig. 2:**
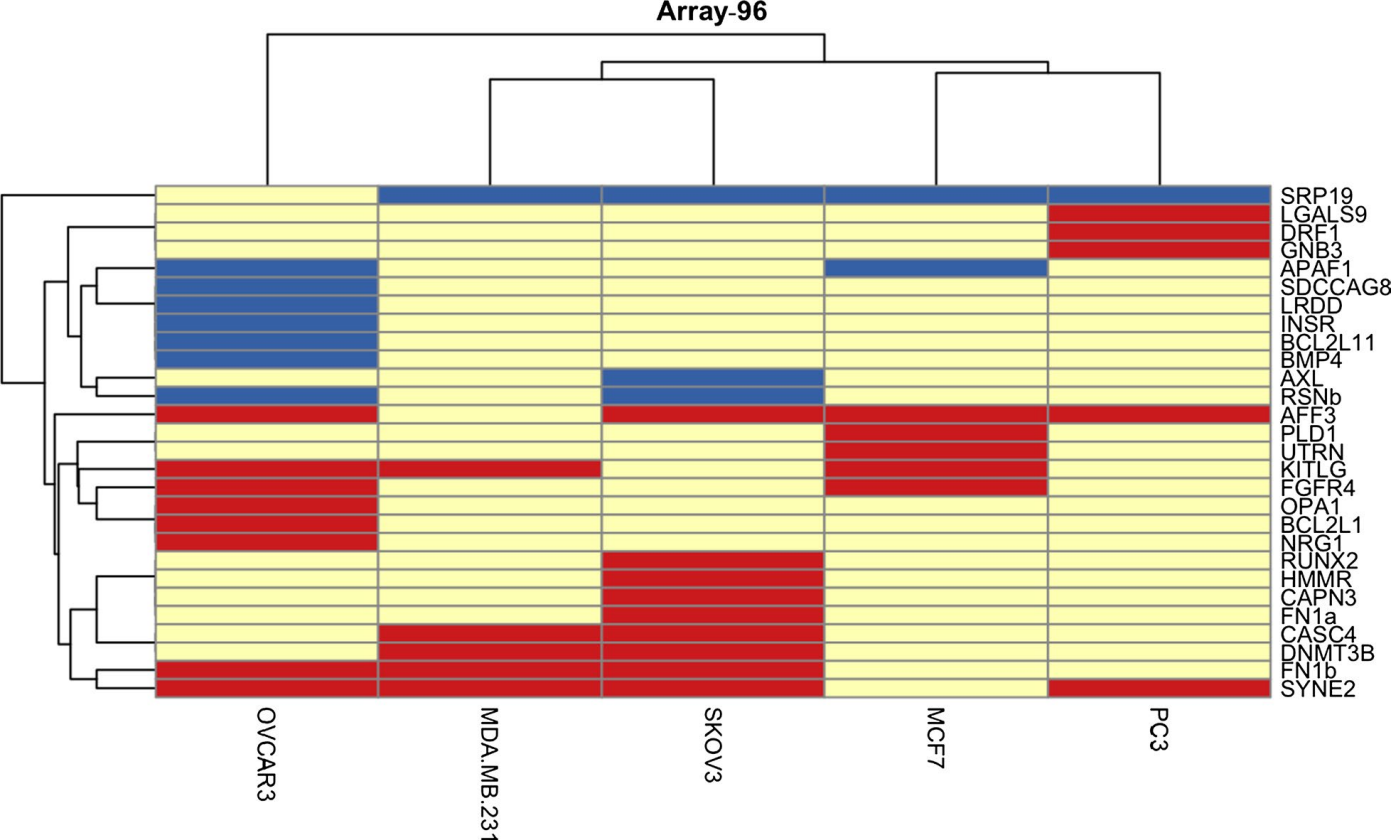
Heatmap and unsupervised hierarchical clustering of genes changed in Array-96 and cell lines used (built using R package ‘pheatmap’). *Each row* represents the gene that had a change in at least one cell line, and *each column* represents the cell line used in the experiment. Preferential exon exclusion shown in *blue*; preferential exon inclusion shown in *red*. The Euclidean distance metric was used for clustering, while the linkage method used was “average” linkage

The results for this screen of 96 ASEs in five cell lines (termed Array-96) demonstrated that 28/96 ASEs (29%) were changed in one or more cell line(s) (http://rnomics.med.usherbrooke.ca/palace?purl=data/related/1811) (Additional file 2), with approximately 18% (5/28 of the genes examined, namely *AFF3*, *FN1*, *KITLG*, *SRP19* and *SYNE2*) being changed in ≥3/5 cell lines (Fig. 2; Additional file 3: Table S1 for an alphabetised listing of ASEs). Knockdown of RBM10 was, therefore, associated with changed ASEs in cancer cell lines.

Almost 68% (19/28) of the changes occurring in one or more cell line(s) involved exon inclusion in the RBM10 KD, while within the cluster of changes common to three or more cell lines (*AFF3*, *FN1b*, *KITLG*, *SRP19* and *SYNE2*) only *SRP19* did not involve exon inclusion in the KD. Refer to Fig. 1 for representative electropherograms of FN1b and KITLG.

Interestingly, both of the ovarian cancer cell lines (OVCAR-3 and SKOV-3) experienced more changes than either of the two breast cancer (MCF-7 and MDA-MB-231) or one prostate cancer (PC-3) cell lines (at 15 and 12 versus 7, 6 and 6, respectively). When we applied unsupervised hierarchical clustering analysis to look for patterns in cell line association, however, while the data showed a good separation of the 28 changes into two major clusters—those consistently experiencing preferential exon exclusion associated with RBM10 KD, and those consistently experiencing preferential exon inclusion associated with RBM10 KD, there was no clustering of changes to either ovarian cancers or breast cancers (Fig. 2). This finding suggests that the regulation of these ASEs by RBM10 was not cancer-type specific.

### 60% of 191 pre-selected ASEs were changed in one or more of four cancer cell lines and one non-cancer cell line upon RBM10 KD

Splicing changes associated with RBM10 KD were next examined in a larger pool (involving 184 genes and 191 ASEs). These units corresponded to a selection of the best events from previous RT-PCR screenings in 10 different cell lines that displayed good yield and reproducibility [22], with 8.3% overlap to the ASEs queried in Array-96. The 191 ASEs were investigated in RNA from the previous RBM10 KDs in the MCF-7, MDA-MB-231, PC-3, SKOV-3 (the same used for Array-96, excluding OVCAR-3) and a new transient RBM10 KD in the non-tumorigenic immortalized fibroblast cell line BJT. This screen was termed Array-191 [http://rnomics.med.usherbrooke.ca/palace?purl=data/related/2493 (Additional file 4)—termed ‘Array-192’ on-line, but the two sets of SMN2 primers were subsequently found to amplify the same ASE]. The percentage of RBM10 KD, in the two transfections of each cell line used in Array-96 and Array-191, is summarized in Table 1.

**Table 1:** Percentage of RBM10 KD in two transfections of each cell line, 96 h post transfection

Cell line	Transfection	% KD RBM10 protein
MCF-7	siRNA1	67
	siRNA2	60
MDA-MB-231	siRNA1	53
	siRNA2	80
NIH:OVCAR-3	siRNA1	55
	siRNA2	80
PC-3	siRNA1	67
	siRNA2	90
SKOV-3	siRNA1	80
	siRNA2	93

**Fig. 3:**
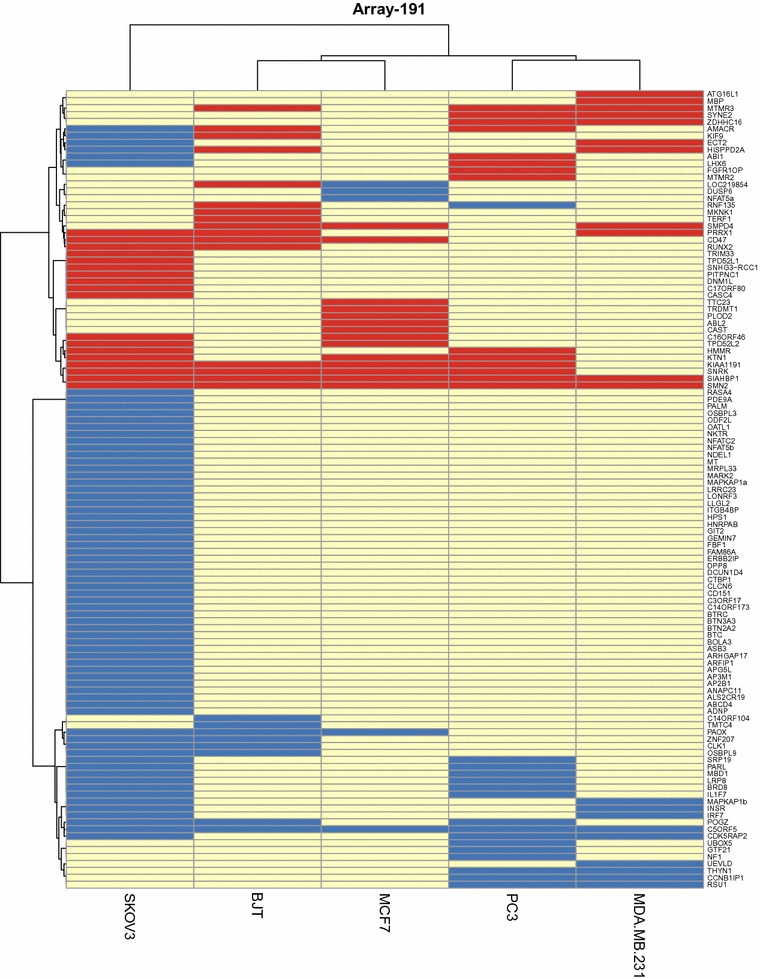
Heatmap and unsupervised hierarchical clustering of genes changed in Array-191 and cell lines used (built using R package ‘pheatmap’). *Each row* represents the gene that had a change in at least one cell line, and *each column* represents the cell line used in the experiment. Preferential exon exclusion shown in *blue*; preferential exon inclusion shown in *red*. The Euclidean distance metric was used for clustering, while the linkage was done using the “ward.D” algorithm

**Fig. 4:**
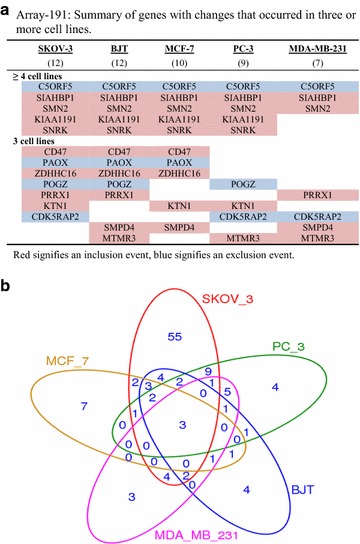
Changes associated with *RBM10* KD in Array-191. **a** Venn diagram showing the relative distribution of changes amongst the five cell lines. Modified from http://faculty.ucr.edu/~tgirke/Documents/R. **b** Summary of changes, by cell line, that occurred in three or more cell lines

As shown in Fig. 3 (and in Additional file 5: Table S2, as an alphabetised listing of ASEs), 115 of the 191 ASEs (60%) were changes in one or more cell line(s), with approximately 12% (14/115) occurring in ≥3/5 cell lines (more clearly illustrated in Fig. 4). 78.5% (11/14) of the knockdown-induced changes occurring in three or more cell lines involved exon inclusion. As seen in Fig. 4, three of the 14 changes (in *C5orf5/FAM13B*, *SIAHBP1* and *SMN2*) occurred in all five cell lines. Refer to Fig. 1 for representative electropherograms of SMN2 and SIAHBP1.

Hierarchical clustering analysis (Fig. 3) revealed that 12.5% (3/24) of the changes occurring in the BJT cells and only one other cancer cell line (in genes *KIF9*, *LOC219854/TMEM218* and *RNF135*) showed a shift in the BJT cells opposite to that which occurred in the cancer cells; however, in each of the three instances, preferential exon exclusion was seen in the cancer cells, but preferential exon inclusion in the BJT cells. Unfortunately, whether or not these differences relate to non-cancer versus cancer, skin versus other tissue type, or fibroblast versus epithelial cell, cannot be determined from the limited number of cell lines examined. The fact that as many changes occurred in the non-cancer cell line (24) as some of the cancer cell lines, e.g., MCF-7 (19), MDA-MB-231 (20) and PC3 (30), did demonstrate, however, that the regulatory effect of RBM10 on alternative splicing was not restricted to cancer cells.

### Confirmation of transient RBM10 KD-associated changes in alternative splicing using a stable RBM10 KD human breast cancer cell line

For a previous study, we had generated a stable RBM10 KD MCF-7 human breast cancer subline [4]. We decided to use this stable RBM10 KD subline to confirm the effect of downregulated RBM10 on specific ASEs.

Focusing on five of the ten changes that occurred specifically in the MCF-7 cells of Array-191, and in two or more of the other cell lines (as listed in Fig. 4a: namely *SIAHBP1/PUF60*, *SMN2*, *KIAA1191*, *POAX* and *SMPD4*), we demonstrated that the alternative splicing changes that occurred in the transient RBM10 KD cells also occurred in the stable RBM10 KD MCF-7 cells [4] (Additional file 6). A relationship between reduced RBM10 expression levels and altered splicing of specific pre-mRNA transcripts was therefore confirmed using a slightly different model system.

### Alternative splicing of RAS-signaling-associated genes is significantly affected by RBM10 KD

We next carried out pathway analysis of the 125 changes that occurred in only those cell lines that were common to both Array-96 and Array-191 (therefore excluding the changes unique to OVCAR-3 in Array-96 and BJT in Array-191), from 259 ASEs examined (Additional file 7: Table S3). The analysis revealed a significant effect of RBM10 KD on *RAS* signaling, since of the 13 *RAS* signaling pathway associated genes that were represented in the combined array ASE pool, five (*ABL2*, *FGF4*, *GNB3*, *KITLG* and *PLD1*) occurring within one hierarchical cluster were changed (*p* = 0.02). Since RAS signaling occurs in all cell types, and is fundamental to many processes—including cell proliferation, differentiation and survival—these results suggested that changes in RBM10 expression levels could impact most, if not all, cell types.

### In human fibroblasts, RBM10 KD correlated with preferential SMN exon 7 inclusion at the RNA level

One of the RBM10 KD-associated changes that was common to all five of the cell lines used in Array-191 occurred in *SMN* pre-mRNA. RBM10 KD consistently correlated with preferential *SMN* exon 7 inclusion, which is associated with the full-length SMN (SMN^FL^) protein. There are two copies of the *SMN* gene in primates, telomeric *SMN1* and the almost identical centromeric *SMN2*. While the two genes encode identical full-length SMN^FL^, *SMN2* predominantly codes for a truncated version, SMN^Δ7^. This truncated version results from a single nucleotide transition, in *SMN2* exon 7, that inhibits splicing of the exon into the pre-mRNA. In people with the disease spinal muscular atrophy (SMA), both alleles of *SMN1* are non-functional, but increased levels of SMN^FL^, usually resulting from *SMN2* gene duplication, correlate with longer survival times [23]. Considering the importance of SMN^FL^ levels to people with SMA, we therefore decided to examine the impact of RBM10 KD on *SMN2* pre-mRNA alternative splicing in fibroblasts from donors with SMA. Based on our splicing array results, we anticipated that RBM10 KD would correlate with an increased ratio of the *SMN2* exon 7-retaining transcript to the exon 7-lacking transcript.

We would like to note here that in Array-191, there were ASEs designated SMN1 and SMN2. The SMN1 ASE related to exon 5, whereas the SMN2 ASE related to exon 7. Since the coding regions of *SMN1* and *SMN2* are almost identical, the primers for all of the SMN ASEs should recognize both *SMN1* and *SMN2* sequences. At the time of primer design for the splicing arrays, alternative splicing of exon 7 was noted as being unique to *SMN2* pre-mRNA (http://rnomics.med.usherbrooke.ca/palace?purl=data/related/2495) (Additional file 1). The current Ensembl database (http://www.emsembl.org), however, shows that alternative splicing of exon 7 occurs in both *SMN1* and *SMN2* pre-mRNAs. That is why, unless we are specifically referring to our results from the SMA fibroblasts (which only contain *SMN2*), we do not refer to the SMN exon 7 ASE as an SMN2 ASE.

**Fig. 5:**
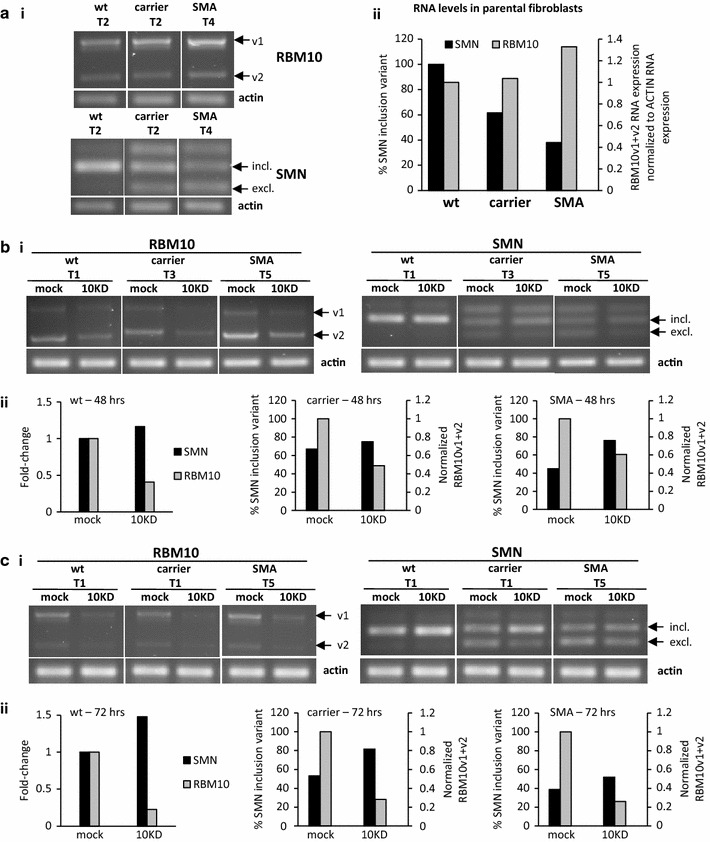
*SMN* RNA expression in human fibroblasts following transient *RBM10* KD. **a** *RBM10* and *SMN* RNA expression in parental fibroblasts: (**i**) representative agarose gel following RT-PCR, and (**ii**) graphed densitometric data from n = 1, representing the clearest data from the indicated transfections T2 and T4. T2 and T4 refer to two different transfections, numbered 2 and 4, respectively. **b** *RBM10* and *SMN* RNA expression in mock transfected (mock) and *RBM10* siRNA transfected (10KD) fibroblasts, 48 h following transfection. **c** *RBM10* and *SMN* RNA expression in mock transfected and *RBM10* siRNA transfected fibroblasts, 72 h following transfection. T1–T5 designate data from five different transfections. The *RBM10* and *SMN* results presented for any particular time point and fibroblast type were from the same transfection

*SMN* exon 7 splicing was examined in control, carrier and SMA Type 1 human fibroblasts. Firstly, we examined endogenous levels of SMN and RBM10 mRNA. As expected, and as shown in Fig. 5a, fibroblasts with wild-type *SMN1* alleles expressed almost exclusively *SMN* exon 7 retaining transcript, fibroblasts from an SMA carrier (which lack one functional *SMN1* allele) expressed both the exon 7 inclusion and exclusion variants, while fibroblasts from an SMA Type 1 patient (which lack both functional *SMN1* alleles) predominantly expressed the exon 7 exclusion variant (encoded by *SMN2*). To note, the additional higher molecular weight SMN band, most clearly observed in the carrier and SMA Type 1 patient samples, is most likely a recently identified splice variant of both SMN1 and SMN2 that excludes exon 7 (54 nts) but includes an exon designated 6B (109 nts), termed SMN6BΔ7 [24]. Also, RBM10v1 and RBM10v2 were expressed in all of the fibroblasts.

As occurred in the splicing arrays, transient inhibition of RBM10 correlated with increased expression of the *SMN* exon 7 inclusion RNA variant and decreased expression of the *SMN* exon 7 exclusion variant, in all of the fibroblasts (Fig. 5b, c). Changes in expression of SMN6BΔ7 were not noted. Data from 48 and 72 h post-RBM10 KD were included to show that in the wt and carrier cells, greater RBM10 KD (at 72 h compared to 48 h) correlated with an increased percentage of the *SMN* inclusion variant. It was because the levels of *SMN* inclusion appeared to correlate with the degree of RBM10 KD, and the degree of RBM10 KD differed between transfections, that the results in Fig. 5 are presented from individual, rather than combined, transfections. Overall, however, our results demonstrate that inhibition of RBM10 expression in human fibroblasts correlates with preferential SMN exon 7 inclusion at the RNA level.

### In human fibroblasts, RBM10 KD correlated with preferential SMN exon 7 inclusion at the protein level

**Fig. 6:**
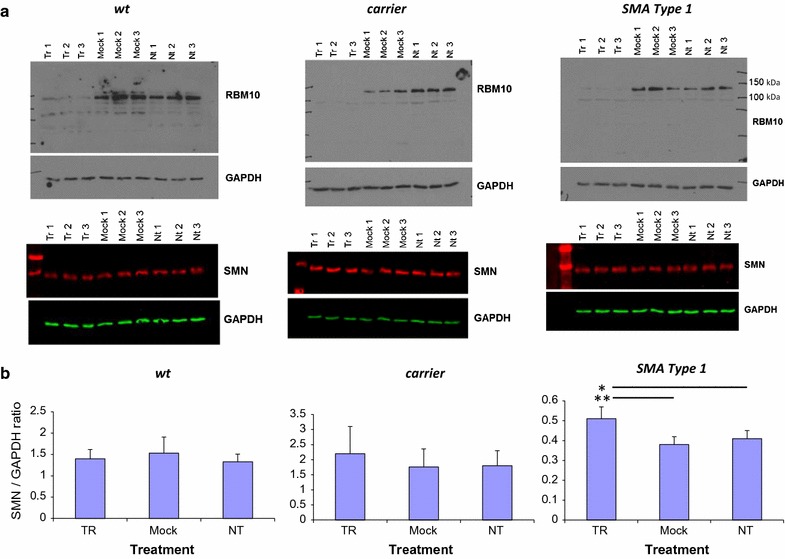
*SMN* protein expression in human fibroblasts following transient *RBM10* KD. Expression was quantified using the Odyssey CLx Imaging System. **a** Three transfections into wt, carrier and SMA Type 1 fibroblasts, with analysis of RBM10 and SMN expression at 96 h post-transfection. The infrared picture for SMN expression in the SMA Type 1 fibroblasts was enhanced to enable visualization of the bands. **b** The ratio of SMN to GAPDH expression for four transfections (n = 4) was graphed. Results were expressed as means and standard errors. Significance was measured using an unpaired Student’s *t* test, where *p = 0.046 and **p = 0.01. *NT* non-transfected, *mock* mock-transfected, *Tr* transfected

Since the level of full-length SMN2 protein in people with SMA is the determinant of SMA severity, we extended our study to see if RBM10 KD correlated with increased SMN^FL^ protein levels, in addition to preferential expression of the exon 7 inclusion RNA variant. As seen in Fig. 6, one SMN band (SMN^FL^) was detected in all the fibroblasts: this result was not unexpected, since the truncated SMN protein (SMN^Δ7^) that is encoded by the SMN exon 7 exclusion variant is an unstable protein [25]. Densitometric analysis of the protein expression data, from four transfections, did, however, reveal a significant increase in the level of SMN^FL^ in the SMA Type 1 fibroblasts, following RBM10 KD (Fig. 6). We did note that, at the protein level, only an almost total lack of RBM10 expression correlated with an increase in SMN^FL^ protein expression levels that was statistically significant, suggesting that even small quantities of RBM10 protein are capable of interfering with *SMN* exon 7 inclusion.

### Confirmation of RBM10 KD-associated preferential SMN exon 7 inclusion using a stable RBM10 KD human breast cancer cell line

**Fig. 7:**
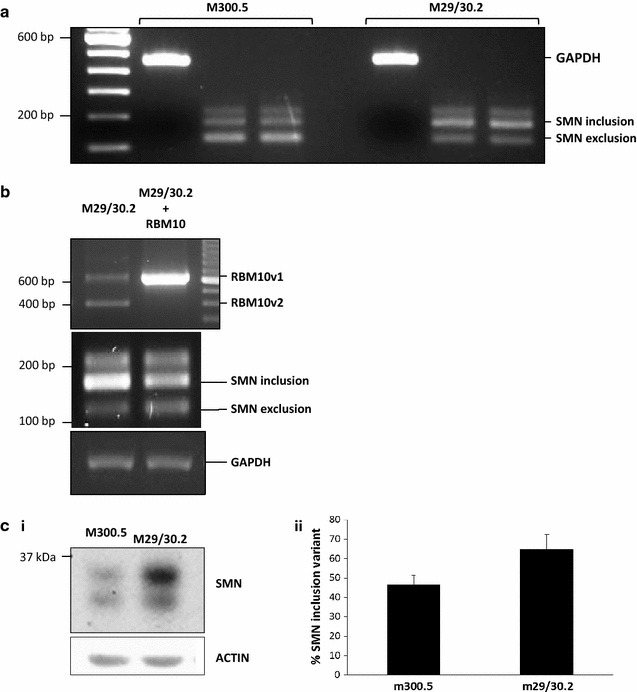
SMN RNA and protein expression in a stable RBM10 KD MCF-7 subline. M300.5 was a stable MCF-7 subline expressing a scrambled shRNA, and M29/30.2 was a stable MCF-7 subline expressing two RBM10 exon 6-specific shRNAs. **a**, **b** RNA expression. **a** *Lane 1* ladder, *lane 2* end-point PCR of M300.5 control using GAPDH primers, *lanes 3* and *4* a duplicate load of an end-point PCR of M300.5 using the SMN primers; *lane 5* blank, *lane 6* end-point PCR of M29/30.2 RBM10 KD using GAPDH primers, *lanes 7* and *8* a duplicate load of an end-point PCR of M29/30.2 using the SMN primers. **b** RNA expression following transient transfection, as previously described [26], of a pcDNA3-based plasmid containing RBM10v1. **c** Protein expression. (**i**) One representative Western blot of SMN protein expression. (**ii**) % SMN^FL^, compared to SMN^Δ7^, from two biological replicates. Results were expressed as means and standard errors

To further characterize the relationship between RBM10 and SMN expression, we decided to use our MCF-7 breast cancer subline with the stable RBM10 KD. Interestingly, both the control and RBM10 KD MCF-7 cells expressed the SMN exon 7 exclusion variant, the exon 7 inclusion variant, and what is likely the exon 6BΔ7 variant (Fig. 7). The control cells, however, showed preferential SMN exon 7 exclusion, whereas the RBM10 KD cells showed preferential SMN exon 7 inclusion (Fig. 7a).

Importantly, re-expression of *RBM10*, following transient transfection of RBM10v1 back into the stable RBM10 KD MCF-7 subline, reverted the splicing change induced by RBM10 KD, resulting in a decreased ratio of *SMN* inclusion to exclusion variant expression (Fig. 7b). This result demonstrates that the altered SMN splicing associated with inhibition of RBM10 is indeed a direct result of the change in RBM10 expression levels.

At the protein level, surprisingly, a second band was observed (Fig. 7ci), a band that could represent an uncharacteristically stable SMN^Δ7^, since the ratio of expression of the inclusion to the exclusion bands increased in the RBM10 KD cells (Fig. 7cii). More surprisingly, in the stable RBM10 KD MCF-7 cells compared to the stable scrambled control subline, total expression of both isoforms increased an average of 2.5-fold (Western blots of two biological replicates, one performed in technical duplicate). These results suggest that long-term, stable, inhibition of RBM10, at least in MCF-7 cells, correlates with not only increased SMN^FL^ expression levels, but stabilization of both SMN isoforms.

## Discussion

When we began these studies, the only identified RBM10 RNA target was the transcript encoding the angiotensin 1 receptor (AT1). In rat vascular smooth muscle cells, RBM10 was shown to bind *AT1* mRNA in the 3′-untranslated region (UTR), resulting in message stabilization and modulation of transcriptional activity [5]. In that study, neither the RBM10 protein binding domain nor the *AT1* RNA target sequence were defined. The findings presented in three more recent RBM10 studies [2, 3, 6] expanded the number of RBM10 targets. The first study, using PAR-CLIP, overexpression, knockdown and RNA-Seq in HEK293 non-transformed, immortalized human embryonic kidney cells, was the first to demonstrate that RBM10 can indeed function to regulate alternative splicing, mediating preferential exon exclusion in a number of pre-mRNAs associated with TARP syndrome and cancer [3]. The second study, using a novel high-throughput screening strategy, demonstrated that RBM10 repressed *Drosophila Dlg4* exon 18 pre-mRNA inclusion, thereby inhibiting synaptogenesis [6]. The third study, which used CLIP-Seq and splicing-sensitive microarrays with HeLa human cervical cancer cells, was the first to identify (1) RBM10 as a regulator of alternative splicing of the NOTCH signaling regulator NUMB, in a cancer cell line, and (2) an RBM10 mRNA target binding motif [2]. Our findings are consistent with data presented in all three publications.

Firstly, our work demonstrates that downregulation of *RBM10* expression correlates with substantial changes in the alternative splicing of a number of pre-mRNAs associated with cancer. For instance, KITLG [also termed “stem cell factor” (SCF) and “mast cell growth factor” (MGF)] is the ligand for the tyrosine kinase receptor c-KIT, involved in growth and development, particularly of haematopoietic and germ cells [27]. An exon 6-containing *KITLG* transcript encodes a precursor transmembrane form of the protein, which can be proteolytically cleaved within the exon 6-encoded region to produce a secreted KITLG isoform [28]. This secreted KITLG isoform supports c-KIT tyrosine kinase activity less efficiently than the transmembrane form, and is mainly involved in stimulating cell proliferation. The exon 6-excluded alternate transcript, associated with wild-type RBM10 levels in our study, is incapable of producing the secreted isoform, since it lacks the metalloprotease cleavage site, and is therefore associated with more active c-KIT tyrosine kinase activity. In our study, *KITLG* exon 6 alternative splicing was investigated in five cancer cell lines. In two breast and one of two ovarian cancer cell lines having RBM10 KD, *KITLG* exon 6 was preferentially included, with normal RBM10 levels associated with exon 6 exclusion. Our findings suggest that, since RBM10 levels are increased in many cancers, *RBM10* may be a potential therapeutic target for those cancers harbouring elevated KITLG (such as many SCLCs [29, 30]).

Secondly, our work supports previous findings that *RBM10* expression is most frequently associated with exon exclusion [2, 3, 6]. Notably, our work confirmed a previously identified association between *RBM10* expression and *SIAHBP1/PUF60* exon 5 exclusion [3]. Both RBM10 and PUF60 are regulators of *DLG4* exon 18 alternative splicing, which itself regulates synaptogenesis [6]. Normal levels of RBM10 are associated with *PUF60* exon 5 exclusion, preferential *DLG4* exon 18 exclusion and synaptic repression [6]. Interestingly, no consensus *RBM10* RNA binding motif, identified in a previous study [2], occurs around *DLG4* exon 18, but does occur in *PUF60* exon 5, suggesting that the regulation of *DLG4* splicing by RBM10 may be an indirect effect that is mediated by the splicing factor PUF60 [31].

Thirdly, our work confirms that changes in *RBM10* expression levels impact both cancer-associated and non-cancer-associated genes. We found that the most significantly affected signaling pathway resulting from *RBM10* inhibition was the *RAS* signaling pathway, which is involved in the regulation of cell growth, differentiation and survival in all cell types. RAS proteins are small GTPases that transmit signals throughout the cell, turning on many genes involved in a range of processes. The five *RAS* pathway associated genes, from Arrays-96 and -191, that were affected by RBM10 inhibition, were a non-receptor tyrosine kinase (*ABL2*), a tyrosine kinase ligand (*KITLG*), a G-protein subunit (*GNB3*), a growth factor receptor (*FGFR4*) and a phospholipase (*PLD1*). Interestingly, recent work [32] showed that RBM10 interacted with the *RAS* signaling associated protein FilGAP, a RAC-specific GTPase activating protein that regulates cell adhesion and migration. Our work therefore supports a potentially fundamental role for RBM10 in signal transduction regulation.

We also identified a novel role for RBM10, as a potential regulator of motor neuron function. One of three consistently altered splicing events that was observed in our arrays occurred in the *SMN* transcript. *SMN* codes for survival of motor neuron (SMN), a protein that is integral to the function of spinal motor neurons [33]. SMN protein is expressed at high levels in the brain, kidney, liver and spinal cord, moderate levels in skeletal and cardiac muscle and low levels in fibroblasts and lymphocytes [34]. In healthy individuals, 80–90% of SMN protein is coded by *SMN1*, since a single nucleotide transition in the *SMN2* gene exon 7 results in exclusion of exon 7 in the majority of expressed RNA, the production of a truncated protein (SMN^Δ7^), and only a small amount of SMN^FL^ produced from that locus. As a consequence, in individuals who have loss of *SMN1* function (as in SMA Type 1 individuals), SMN^FL^ encoded by *SMN2* cannot entirely compensate. Reduced SMN^FL^ leads to a progressive degeneration of motor neurons, spinal muscular atrophy and eventual pulmonary failure [35]. The less SMN^FL^ produced (related to *SMN2* gene copy number), the more severe the disease [36]. Regulators of *SMN2* exon 7 alternative splicing include hnRNP G, hnRNP M, hnRNP Q, PSF, Sam68, SRSF1, SRSF9 and TRA2β [37–43] and another RBM10 target, PUF60 [31]. Binding of RBM10 to *SMN* pre-mRNA was not found using PAR-CLIP in HEK293 cells [3], so any potential influence of RBM10 on *SMN* alternative splicing might be indirect. As suggested for the regulation of *DLG4* alternative splicing (mentioned above), RBM10 associated regulation of *SMN2* alternative splicing may occur through PUF60, since changes in *PUF60* expression levels have previously been shown (in HeLa cells) to alter *SMN2* exon 7 splicing [31].

## Conclusions

In our study, because all of the cell lines examined showed increased expression of RNA encoding SMN^FL^ following downregulation of RBM10, *RBM10* may be a potential target for SMA therapy. The usefulness of our findings, with regards to SMA, would depend on the identification of an RBM10 antagonist that could specifically target motor neurons important to SMA, since downregulation of *RBM10* (particularly during embryonic development) is the causative agent for TARP syndrome [9, 10]. The role played by RBM10 in TARP syndrome is unknown, but likely involves its ability to function as a splicing regulator [3]. The findings we report here suggest that TARP syndrome might be associated with not only dysfunctional RBM10 but elevated levels of either or both isoforms of SMN2. Since SMN^FL^ is also involved in RNA processing, the relationship between *RBM10* downregulation, increased production of SMN^FL^ and RNA processing in both TARP syndrome and SMA warrants further investigation.

## Additional files

**Additional file 1.** http://rnomics.med.usherbrooke.ca/palace?purl=pcrreactiongroup/list/299. Genomics Platform-Palace: Overview Page.

**Additional file 2.** http://rnomics.med.usherbrooke.ca/palace?purl=data/related/1811. Array-96--RBM10 Knockdowns in MCF-7, MDA-MB-231, SKOV-3, OVCAR-3 and PC-3.

**Additional file 3: Table S1.** Array-96: List of genes with changes, by cell line.

**Additional file 4.** http://rnomics.med.usherbrooke.ca/palace?purl=data/related/2493. Array-191--RBM10 Knockdowns in BJ-TIELF, MCF-7, MDA-MB-231, SKOV-3, and PC-3.

**Additional file 5: Table S2.** Array-191: List of genes with changes, by cell line.

**Additional file 6: Figure S1.** Verification of splicing changes in a stable MCF-7 RBM10 KD.

**Additional file 7: Table S3.** List of changes that occurred in cell lines common to both Array-96 and Arrar-191, and the GeneCard database designation used for pathway analysis.

## Abbreviations

RBM10: RNA Binding Motif 10
SMN: survival of motor neuron
SMA: spinal muscular atrophy
ASE: alternative splicing event
KD: knockdown
Δψ: percent splicing index change
SMN^FL^: full-length SMN protein
SMN^Δ7^: SMN protein with an exon 7 deletion
