# Supplementary material for: Splicing arrays reveal novel RBM10 targets, including SMN2 pre-mRNA
Source: BMC Mol Biol. 2017 Jul 20;18:19. doi: 10.1186/s12867-017-0096-x (PMC5520337; doi:10.1186/s12867-017-0096-x)
Supplement: Supplementary file 3 — Additional file 3: Table S1. Array-96: List of genes with changes, by cell line. [file 12867_2017_96_MOESM3_ESM.pdf]

Additional file 3

Supplemental Table 1. Array-96: List of genes with changes, by cell line.

<table>
<thead>
<tr><th></th><th><u>MCF-7</u>
(7)</th><th><u>MDA-MB-231</u>
(6)</th><th><u>OVCAR-3</u>
(15)</th><th><u>SKOV-3</u>
(12)</th><th><u>PC-3</u>
(6)</th></tr>
</thead>
<tbody>
<tr><td>1. AFF3</td><td bgcolor="#e6b8b7"></td><td></td><td bgcolor="#e6b8b7"></td><td bgcolor="#e6b8b7"></td><td bgcolor="#e6b8b7"></td></tr>
<tr><td>2. APAF1</td><td bgcolor="#b8cce4"></td><td></td><td bgcolor="#b8cce4"></td><td></td><td></td></tr>
<tr><td>3. AXL</td><td></td><td></td><td></td><td bgcolor="#b8cce4"></td><td></td></tr>
<tr><td>4. BCL2L1</td><td></td><td></td><td bgcolor="#e6b8b7"></td><td></td><td></td></tr>
<tr><td>5. BCL2L11</td><td></td><td></td><td bgcolor="#b8cce4"></td><td></td><td></td></tr>
<tr><td>6. BMP4</td><td></td><td></td><td bgcolor="#b8cce4"></td><td></td><td></td></tr>
<tr><td>7. CAPN3</td><td></td><td></td><td></td><td bgcolor="#e6b8b7"></td><td></td></tr>
<tr><td>8. CASC4</td><td></td><td bgcolor="#e6b8b7"></td><td></td><td bgcolor="#e6b8b7"></td><td></td></tr>
<tr><td>9. DNMT3B</td><td></td><td bgcolor="#e6b8b7"></td><td></td><td bgcolor="#e6b8b7"></td><td></td></tr>
<tr><td>10. DRF1</td><td></td><td></td><td></td><td></td><td bgcolor="#e6b8b7"></td></tr>
<tr><td>11. FGFR4</td><td bgcolor="#e6b8b7"></td><td></td><td bgcolor="#e6b8b7"></td><td></td><td></td></tr>
<tr><td>12. FN1a</td><td></td><td></td><td></td><td bgcolor="#e6b8b7"></td><td></td></tr>
<tr><td>13. FN1b</td><td></td><td bgcolor="#e6b8b7"></td><td bgcolor="#e6b8b7"></td><td bgcolor="#e6b8b7"></td><td></td></tr>
<tr><td>14. GNB3</td><td></td><td></td><td></td><td></td><td bgcolor="#e6b8b7"></td></tr>
<tr><td>15. HMMR</td><td></td><td></td><td></td><td bgcolor="#e6b8b7"></td><td></td></tr>
<tr><td>16. INSR</td><td></td><td></td><td bgcolor="#b8cce4"></td><td></td><td></td></tr>
<tr><td>17. KITLG</td><td bgcolor="#e6b8b7"></td><td bgcolor="#e6b8b7"></td><td bgcolor="#e6b8b7"></td><td></td><td></td></tr>
<tr><td>18. LGALS9</td><td></td><td></td><td></td><td></td><td bgcolor="#e6b8b7"></td></tr>
<tr><td>19. LRDD</td><td></td><td></td><td bgcolor="#b8cce4"></td><td></td><td></td></tr>
<tr><td>20. NRG1</td><td></td><td></td><td bgcolor="#e6b8b7"></td><td></td><td></td></tr>
<tr><td>21. OPA1</td><td></td><td></td><td bgcolor="#e6b8b7"></td><td></td><td></td></tr>
<tr><td>22. PLD1</td><td bgcolor="#e6b8b7"></td><td></td><td></td><td></td><td></td></tr>
<tr><td>23. RSNb</td><td></td><td></td><td bgcolor="#b8cce4"></td><td bgcolor="#b8cce4"></td><td></td></tr>
<tr><td>24. RUNX2</td><td></td><td></td><td></td><td bgcolor="#e6b8b7"></td><td></td></tr>
<tr><td>25. SDCCAG8</td><td></td><td></td><td bgcolor="#b8cce4"></td><td></td><td></td></tr>
<tr><td>26. SRP19</td><td bgcolor="#b8cce4"></td><td bgcolor="#b8cce4"></td><td></td><td bgcolor="#b8cce4"></td><td bgcolor="#b8cce4"></td></tr>
<tr><td>27. SYNE2</td><td></td><td bgcolor="#e6b8b7"></td><td bgcolor="#e6b8b7"></td><td bgcolor="#e6b8b7"></td><td bgcolor="#e6b8b7"></td></tr>
<tr><td>28. UTRN</td><td bgcolor="#e6b8b7"></td><td></td><td></td><td></td><td></td></tr>
</tbody>
</table>

( ) = total number of changes per cell line.
Red signifies an inclusion event.
Blue signifies an exclusion event.
