# Supplementary material for: Splicing arrays reveal novel RBM10 targets, including SMN2 pre-mRNA
Source: BMC Mol Biol. 2017 Jul 20;18:19. doi: 10.1186/s12867-017-0096-x (PMC5520337; doi:10.1186/s12867-017-0096-x)
Supplement: Supplementary file 5 — Additional file 5: Table S2. Array-191: List of genes with changes, by cell line. [file 12867_2017_96_MOESM5_ESM.pdf]

Additional file 5

Supplemental Table 2. Array-191: List of genes with changes, by cell line.

	SKOV-3 (87)	PC-3 (30)	BJT (24)	MDA-MB-231 (20)	MCF-7 (20)	Alias
1.	ABCD4					
2.	ABI1	ABI1				
3.					ABL2	
4.	ADNP					
5.	ALS2CR19					/PARD3B
6.	AMACR	AMACR	AMACR			
7.	ANAPC11					
8.	AP2B1					
9.	AP3M1					
10.	APG5L					/ATG5
11.	ARFIP1					
12.	ARHGAP17					
13.	ASB3					/GPR75-ASB3
14.				ATG16L1		
15.	BOLA3					
16.	BRD8	BRD8				
17.	BTC					
18.	BTN2A2					
19.	BTN3A3					
20.	BTRC					
21.			C14ORF104			
22.	C14ORF173					/INF2
23.	C16ORF46				C16ORF46	
24.	C17ORF80					
25.	C3ORF17					/AKIP1
26.	C5ORF5	C5ORF5	C5ORF5	C5ORF5	C5ORF5	/FAM13B
27.	CASC4					
28.					CAST	
29.		CCNB1IP1		CCNB1IP1		
30.	CD47		CD47		CD47	
31.	CD151					
32.	CDK5RAP2	CDK5RAP2		CDK5RAP2		
33.	CLCN6					
34.	CLK1		CLK1			
35.	CTBP1					
36.	DCUN1D4					
37.	DNM1L					
38.	DPP8					
39.					DUSP6	
40.	ECT2			ECT2		
41.	ERBB2IP					
42.	FAM86A					
43.	FBF1					
44.		FGFR1OP				
45.	GEMIN7					
46.	GIT2					
47.		GTF21				
48.	HISPPD2A		HISPPD2A	HISPPD2A		/PPIP5K1
49.	HMMR	HMMR				
50.	HNRPAB					/HNRNPAB
51.	HPS1					
52.	IL1F7	IL1F7				/IL37
53.	INSR			INSR		
54.	IRF7			IRF7		
55.	ITGB4BP					/EIF6
56.	KIAA1191	KIAA1191	KIAA1191		KIAA1191	
57.	KIF9		KIF9			
58.	KTN1	KTN1			KTN1	
59.	LHX6	LHX6				
60.	LLGL2					
61.			LOC219854		LOC219854	
62.	LONRF3					
63.	LRP8	LRP8				
64.	LRRC23					
65.	MAPKAP1a					
66.	MAPKAP1b			MAPKAP1b		/MAPKAP1
67.	MARK2					
68.	MBD1	MBD1				
69.				MBP		
70.			MKNK1			
71.	MRPL33					
72.	MT					/MMP16
73.		MTMR2				
74.		MTMR3	MTMR3	MTMR3		
75.	NDEL1					
76.		NF1				
77.					NFAT5a	
78.	NFAT5b					/NFAT5
79.	NFATC2					
80.	NKTR					
81.	OATL1					/TBC1D25
82.	ODF2L					
83.	OSBPL3					
84.	OSBPL9		OSBPL9			
85.	PALM					
86.	PAOX		PAOX		PAOX	
87.	PARL	PARL				
88.	PDE9A					
89.	PITPNC1					
90.					PLOD2	
91.	POGZ	POGZ	POGZ			
92.	PRRX1		PRRX1	PRRX1		
93.	RASA4					
94.		RNF135	RNF135			
95.		RSU1		RSU1		
96.	RUNX2		RUNX2			
97.	SIAHBP1	SIAHBP1	SIAHBP1	SIAHBP1	SIAHBP1	/PUF60
98.		SMN2	SMN2	SMN2		
*	SMN2	SMN2	SMN2		SMN2	
99.			SMPD4	SMPD4	SMPD4	
100.	SNHG3-RCC1					/RCC1
101.	SNRK	SNRK	SNRK		SNRK	
102.	SRP19	SRP19				
103.		SYNE2		SYNE2		
104.			TERF1			
105.		THYN1		THYN1		
106.			TMTC4			
107.	TPD52L1					
108.	TPD52L2				TPD52L2	
109.					TRDMT1	
110.	TRIM33					
111.					TTC23	
112.		UBOX5				
113.				UEVLD		
114.	ZDHHC16		ZDHHC16		ZDHHC16	
115.		ZNF207		ZNF207		

* Same ASE but different primer set.

Red signifies an inclusion event.

Blue signifies an exclusion event.

( ) = total # of changes in Array-191, per cell line.
