# Supplementary material for: Splicing arrays reveal novel RBM10 targets, including SMN2 pre-mRNA
Source: BMC Mol Biol. 2017 Jul 20;18:19. doi: 10.1186/s12867-017-0096-x (PMC5520337; doi:10.1186/s12867-017-0096-x)
Supplement: Supplementary file 6 — Additional file 6: Figure S1. Verification of splicing changes in a stable MCF-7 RBM10 KD. [file 12867_2017_96_MOESM6_ESM.pdf]

Additional file 6

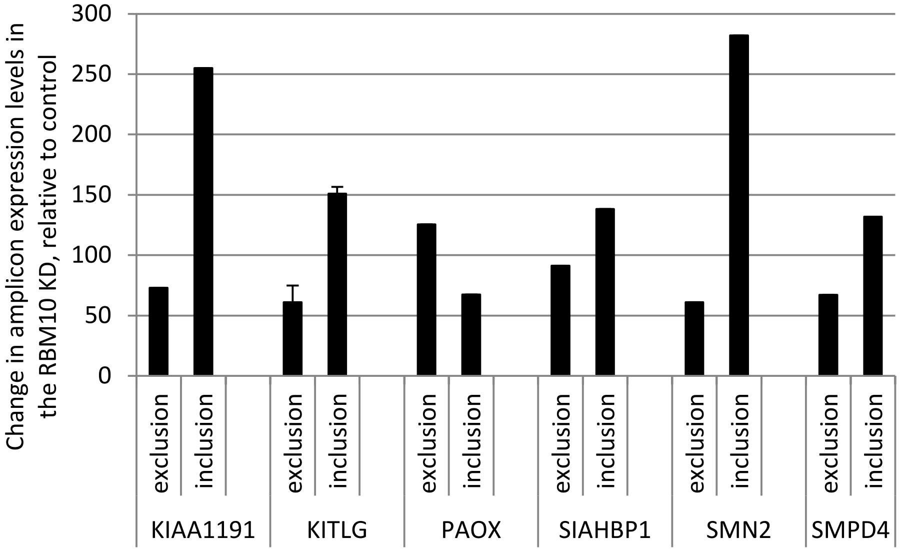

Supplemental Fig. 1 Verification of splicing changes in a stable MDF-7 RBM10 KD. A stable clonal RBM10 KD MCF-7 subline with 80% RBM10 protein KD was used to examine a subset of the changes identified in Array-96 and Array-191. Experimentally, this was accomplished using end-point PCR and the same primer pairs that were used to generate the capillary electrophoresis-monitored ASEs. Graphed data are from densitometric analyses of amplicons visualized on agarose gels. N of 1: SIAHBP, 2: KIAA1191, KITLG or 3: POAX, SMPD4. Error bars represent standard error.
