# Supplementary material for: Splicing arrays reveal novel RBM10 targets, including SMN2 pre-mRNA
Source: BMC Mol Biol. 2017 Jul 20;18:19. doi: 10.1186/s12867-017-0096-x (PMC5520337; doi:10.1186/s12867-017-0096-x)
Supplement: Supplementary file 7 — Additional file 7: Table S3. List of changes that occurred in cell lines common to both Array-96 and Arrar-191, and the GeneCard database designation used for pathway analysis. [file 12867_2017_96_MOESM7_ESM.pdf]

Additional file 7

Supplemental Table 3. List of changes that occurred in cell lines common to both Array-96 and Array-191, and the GeneCard database designation used for pathway analysis.

Array - 96	Array - 192	Corresponding GeneCard Designation	# of ASEs
	ABCD4	ABCD4	1
	ABI1	ABI1	2
	ABL2	ABL2	3
	ADNP	ADNP	4
AFF3		AFF3	5
	ALS2CR19	PARD3B	6
	AMACR	AMACR	7
	ANAPC11	ANAPC11	8
	AP2B1	AP2B1	9
	AP3M1	AP3M1	10
APAF1		APAF1	11
	APG5L	ATG5	12
	ARFIP1	ARFIP1	13
	ARHGAP17	ARHGAP17	14
	ASB3	GPR75-ASB3	15
	ATG16L1	ATG16L1	16
AXL		AXL	17
	BOLA3	BOLA3	18
	BRD8a	BRD8	19
	BTC	BTC	20
	BTN2A2	BTN2A2	21
	BTN3A3	BTN3A3	22
	BTRC	BTRC	23
	C3orf17	C3orf17	24
	C5orf5	FAM13B	25
	C14orf173	INF2	26
	C16orf46	C16orf46	27
	C17orf80	C17orf80	28
CAPN3		CAPN3	29
CASC4	CASC4a	CASC4	30
	CAST	CAST	31
	CCNB1IP1	CCNB1IP1	32
	CD151	CD151	33
	CD47	CD47	34
	CDK5RAP2	CDK5RAP2	35
	CLCN6	CLCN6	36
	CLK1	CLK1	37
	CTBP1	CTBP1	38
	DCUN1D4	DCUN1D4	39
	DNM1L	DNM1L	40
DNMT3B		DNMT3B	41
	DPP8	DPP8	42
DRF1		DBF4B	43
	DUSP6	DUSP6	44
	ECT2	ECT2	45
	ERBB2IP	ERBB2IP	46
	FAM86A	FAM86A	47
	FBF1	FBF1	48
	FGFR1OP	FGFR1OP	49
FGFR4		FGFR4	50
FN1a		FN1	51
FN1b			52
	GEMIN7	GEMIN7	53

	GIT2	GIT2	54
GNB3		GNB3	55
	GTF2I	GTF2I	56
	HISPPD2A	PPIP5K1	57
HMMR	HMMR	HMMR	58
	HNRPAB	HNRNPAB	59
	HPS1	HPS1	60
	IL1F7	IL37	61
	INSR	INSR	62
	IRF7	IRF7	63
	ITGB4BP	EIF6	64
	KIAA1191	KIAA1191	65
	KIF9	KIF9	66
KITLG		KITLG	67
	KTN1a	KTN1	68
LGALS9		LGALS9	69
	LHX6	LHX6	70
	LLGL2	LLGL2	71
	LOC219854	TMEM218	72
	LONRF3	LONRF3	73
	LRP8	LRP8	74
	LRRC23	LRRC23	75
	MAPKAP1a	MAPKAP1	76
	MAPKAP1b		77
	MARK2	MARK2	78
	MBD1	MBD1	79
	MBP	MBP	80
	MRPL33	MRPL33	81
	MT	MCAT	82
	MTMR2	MTMR2	83
	MTMR3	MTMR3	84
	NDEL1	NDEL1	85
	NF1	NF1	86
	NFAT5a	NFAT5	87
	NFAT5b		88
	NFATC2	NFATC2	89
	NKTR	NKTR	90
	OATL1	TBC1D25	91
	ODF2L	ODF2L	92
	OSBPL3	OSBPL3	93
	OSBPL9	OSBPL9	94
	PALM	PALM	95
	PAOX	PAOX	96
	PARL	PARL	97
	PDE9A	PDE9A	98
	PITPNC1	PITPNC1	99
PLD1		PLD1	100
	PLOD2	PLOD2	101
	POGZ	POGZ	102
	PRRX1	PRRX1	103
	RASA4	RASA4	104
	RNF135	RNF135	105
RSNb		RSN	106
	RSU1	RSU1	107
RUNX2	RUNX2	RUNX2	108
	SIAHBP1	PUF60	109
	SMN2	SMN2	110
	SMPD4	SMPD4	111
	SNHG3-RCC1	RCC1	112
	SNRK	SNRK	113
SRP19	SRP19	SRP19	114
	THYN1	THYN1	115
	TPD52L1	TPD52L1	116

	TPD52L2	TPD52L2	117
	TRDMT1	TRDMT1	118
	TRIM33	TRIM33	119
	TTC23	TTC23	120
	UBOX5	UBOX5	121
	UEVLD	UEVLD	122
UTRN		UTRN	123
	ZDHHC16	ZDHHC16	124
	ZNF207	ZNF207	125
